# Drug shortages in European countries: a trade-off between market attractiveness and cost containment?

**DOI:** 10.1186/1472-6963-14-438

**Published:** 2014-09-26

**Authors:** Kim Pauwels, Isabelle Huys, Minne Casteels, Steven Simoens

**Affiliations:** KU Leuven Department of Pharmaceutical and Pharmacological Sciences, Onderwijs en Navorsing 2, Herestraat 49, P.O. Box 521, 3000 Leuven, Belgium

**Keywords:** Drug shortage, Drug supply, Health policy

## Abstract

**Background:**

Drug shortages are a global problem. While extensively studied in the United States, numbers about drug shortages in European countries are scarce. This study aims to collect and present data about drug shortages in European countries.

**Methods:**

A reporting template for the collection of data about drug shortages was designed based on a literature search. Countries offering a reporting system for drug shortages such as Belgium, the Netherlands, England, Italy, France, Germany and Spain were included in this study. Data about the characteristics of the drugs in shortage and the causes of the shortage were collected from publicly available online reporting systems. Descriptive analyses were performed.

**Results:**

Drug shortages included in the considered reporting systems can be characterized as branded, oral drugs that affect different disease domains. When considering essential medicines and oncology drugs, generic injectables are more involved. Causes for drug shortages are largely underreported. In case the cause is known, production problems take the lead.

**Conclusions:**

Reporting of drug shortages in Europe needs to be standardized and more transparency about the reasons for drug shortage is required to investigate the problem. A link between production problems and market attractiveness and market capacity is recognized to be at the root of drug shortages in U.S. Such insights are highly lacking in Europe. Monitoring of the effect of national and European health policies on the sustainability of the drug market is required to present fundamental solutions and to tackle the problem of drug shortages in Europe.

## Background

Drug shortages are recognized as a global problem by the World Health Organization (WHO) [1]. Drug shortages can be defined as a shortcoming in the supply of a medicinal product which makes it impossible for suppliers to meet the demand for the product at patient level. It affects all stakeholders of the health care system such as patients, pharmacists, clinicians, the pharmaceutical industry and policy makers. Patients need to switch to an alternative therapy which may cause adverse effects, increased incidence of medication errors and disease progression [2, 3]. Additionally drug shortages will bring along monetary costs due to switchovers to alternative therapies and increased workload to manage the shortage [2, 3]. The origin of a drug shortage problem can possibly be located at the supply and/or demand side. At the level of drug supply, manufacturing difficulties, unavailability of raw materials and natural disasters are reported [4, 5]. Cessation of the production of a drug can also be an economic decision of the manufacturer [5]. Additionally, the supply side can be influenced by policy measures such as restricted drug production or allocation and quality requirements [6]. At the demand side, causes of drug shortage proposed in literature are unexpected increase in demand, unforeseen shifts in clinical practice and parallel trade [6].

Drug shortages affect the health care system in the United States (U.S.) more than ever before. Numbers of reported shortages increased from 60 drug shortages in 2005 to more than 200 reported drug shortages in 2010 [3, 4, 6]. Since 2011, the U.S. Food and Drug Administration (FDA) has taken initiatives to prevent and mitigate the impact of drug shortages in the U.S. Although pharmaceutical manufacturers cannot be forced to produce (an additional amount of) a particular drug, FDA aims to provide knowledge about drug shortages to health care providers in advance of the shortage [5]. Therefore, manufacturers are mandated to notify the FDA about potential discontinuations in supply of drugs for serious or life-threatening conditions [6]. A more extensive database of U.S. drug shortages is maintained by the American Society of Health-system Pharmacists (ASHP). Monitoring of drug shortages does not only promote early anticipation of drug shortages by health care workers, it does also provide a source for extensive data analysis. It has been reported that U.S. drug shortages are mostly affecting the generic injectable market with majority of drugs in shortage indicated for oncology indications [5–7].

Although exact figures about drug shortages in Europe are lacking, the emerging number of reports and studies about drug shortages in European countries leave no doubt that the problem is also affecting the European drug market [8–14]. While the consequences of drug shortages are likely to be comparable between U.S. and Europe, the causes are expected to depend on specific features of the health care market, determined by the regulatory framework and health policy applied in a particular country. There is no centralized database collecting information about drug shortages in European countries. Given that understanding of the “what” and “why” is essential to present fundamental solutions, the goal of this study is to collect and present data about drug shortages in European countries. By way of a case study, special focus will go to oncology drugs as shortages in the U.S. are mostly occurring in this disease domain, while the impact in Europe is unknown. Shortages of oncology drugs pose a special challenge due to the number of patients involved, the multiple indications that one oncology drug often has and the narrow therapeutic window of oncology therapies in which small adjustments in dose can have a tremendous effect.

## Methods

A reporting template was designed to collect the data about drug shortages in Europe in a standardized, systematic and reproducible way. The features included in this reporting template aim to cover all aspects involved in typology and causes of drug shortages, based on a literature search. The literature was searched through Pubmed and Embase between May and June 2013 by a combination of following search terms: drug purchasing, drug marketing, drug supply, drug shortage, drug crisis, drug in short supply, stock problem. Articles concerning drug shortages, published in English between 2010 and 2013, were selected to ensure up-to-date information. Further, websites of the following international and European health care associations were consulted: World Health Organization (WHO), European Medicines Agency (EMA), European Generics Association (EGA), European Association of Hospital Pharmacists (EAHP), European Association of Pharmaceutical Full-line Wholesalers (GIRP), International Society for Pharmaco economics and Outcomes Research (ISPOR), Food and Drug Administration, American Society of Health System Pharmacists (ASHP), and European Federation of Pharmaceutical Industries and Associations (EFPIA). Next, websites of national health authorities of European countries were checked for the presence of a reporting system concerning drug shortages. Based on this first survey, Belgium, the Netherlands, England, Italy, Germany, Spain and France were selected for inclusion in this study based on the presence of a reporting system for drug shortages (Table 1). The reporting systems presented in Table 1 were consulted in May 3013 and the designed reporting template was used to collect data about drug shortages.

**Table 1:** **Data sources consulted to complete the reporting template and scope of the reporting systems**

Country	National shortage reporting system	Reporter	Drugs included in reporting system	National drug formulary for hospitals or community pharmacy consulted to complete reporting template
Belgium	Federal Agency for Medicines and Health Products (FAGG)	Marketing authorization holders	Drug for which unavailability cause a risk for public health and have no therapeutic alternative	Belgian Centre for Pharmacotherapeutic Information (BCFI)
	Farmaco-contingentering.be	Pharmacists	Products subjected to supply quota	
The Netherlands	Farmanco.nl	Marketing authorization holders, wholesalers, pharmacists	Unlimited	Geneesmiddelenrepertorium.nl
England	Pharmaceutical Services Negotiation Committee (PSNC)	Pharmacists	Products for community pharmacy only	Eudrapharm
Italy	Italian Medicines Agency (AIFA)	Marketing authorization holders, health care providers, health departments, patients or associations	Unlimited	Italian Drug Formulary (AIFA)
				Eudrapharm
Germany	Federal Institute for Drugs and Medical Devices (Bfarm)	Marketing authorization holders	Products for treatment of threatening or serious diseases for which no alternatives are available	Federal Institute for Drugs and Medical Devices (Bfarm)
				Eudrapharm
Spain	Spanish Agency for Medicines and Health Products (aemps)	Marketing authorization holders or health authorities of autonomous communities	Unlimited	Spanish Agency for Medicines and Health Products (aemps)
France	National Agency for Safety of Drugs and Health Products (ansm)	Pharmacists and hospitals	Drug for which unavailability cause a risk for public health and have no therapeutic alternative	National Agency for Safety of Drugs and Health Products (ansm)

Before the start of the analyses the data were checked for duplicates and the data set was filtered to include prescription drugs only. Additionally, oncology drugs were selected based on the Anatomic Therapeutic Chemical Classification System (ATC) code L01 and L03 for a sub-analysis. Given that literature about typology of drug shortages is mainly limited to U.S. studies, a sub-analysis was also performed for drugs defined as essential medicines by the WHO to increase comparability with U.S. and thus the literature [15]. Descriptive statistics on the characteristics and causes of drugs shortages were calculated.

## Results

**Figure 1:**
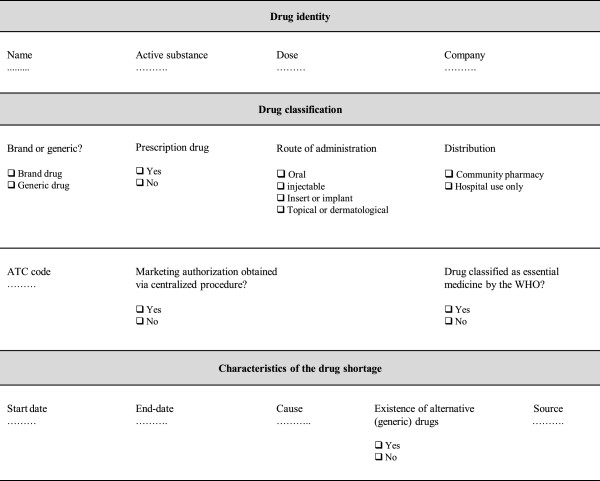
**Reporting template for drug shortages.**

The reporting template was designed to collect the following information (Figure 1): country of shortage, name of the drug, active substance, dose(s) of the drug affected by shortage, route of administration, listed as essential medicine by the WHO or not, marketing authorization obtained via centralized procedure or not, branded or generic drug, ATC code according to the WHO Collaborating Centre for Drug Statistics Methodology [16], requirement of prescription, drug used in hospital setting only or community pharmacy, start date of the shortage, end date of the shortage, concerned company, cause, existence of alternative (generic) drugs, source of information. The route of administration was classified in four groups: oral, injectable, insert or implant, topical or dermatological. The causes were fully described in the reporting template during the collection of data for this study.

Table 1 presents the sources consulted to collect the data for this study. The different reporting systems consulted in this study each rely on particular reporters to obtain the information and the scope of drugs presented in the reporting system differs between different reporting systems. The reporter and scope of the reporting system is presented in Table 1.

**Figure 2:**
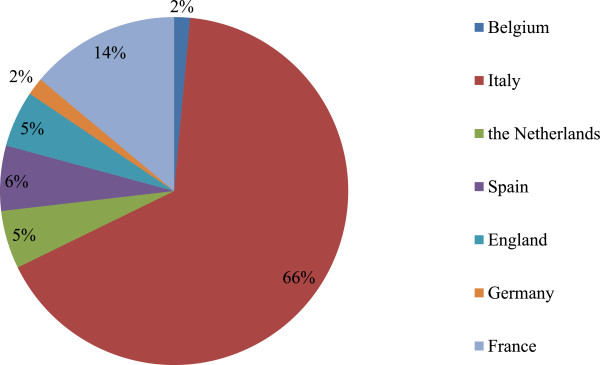
**Proportion of reported shortages per country.** n = 161.

Information about 671 drug shortages was collected. Figure 2 shows the proportion of shortages reported in each country considered in this study. In France, the reporting system notified one shortage that happened in 2005 and one shortage recorded in 2009. In the Netherlands, the reporting system mentioned a shortage that occurred in 2007. Start dates of all other shortages included in the reporting systems are between January 2010 and August 2013. In five percent of the cases (31/671), information about the start data was however not reported. In 68% (459/671) information about the predicted end date of shortage was not available. Seventy three percent of the shortages for which information about the predicted end of the shortage (154/212) was available, was presented as a permanent cessation.

For those cases for which both the start date as well as the end date were available, the median duration of the shortage was 139 days (n = 54). Based on available information about start date and end date of the shortages, on June 1^st^ 2013, 21 shortages were still ongoing. In 6% of the cases for which the start date was available (38/640) a start date after June 1^st^ 2013 was mentioned and the shortages was thus announced in advance.

Nine percent (61/671) concern drugs that obtained marketing authorization through the centralized procedure of EMA, involving 32 different products. EMA reported the shortage for only seven of these products.

**Figure 3:**
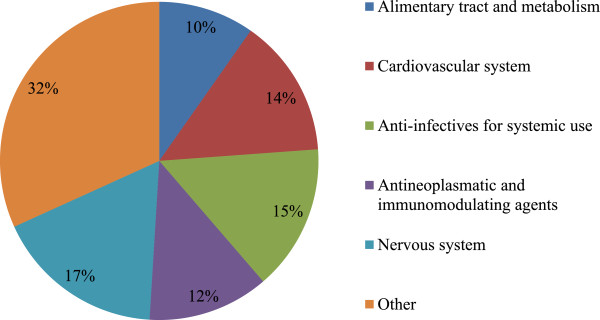
**Proportion of reported drugs per Anatomic Therapeutic Chemical Class.** Drugs classified in the group “other” include antiparasitic products, insecticides and repellents, products for the respiratory system, products for the sensory organs, drugs affecting blood and blood forming organs and dermatologicals. These are all grouped for the sake of clarity. n = 161.

Over 50% of reported shortages affected medicines included in one of the five major ATC classes: cardiovascular system, anti-infectives for systemic use, antineoplastic and immune modulating agents and the nervous system (Figure 3). Thirty percent of the reported shortages (200/671) affected drugs defined as essential medicines by the WHO. Further results additionally focus on oncology drugs and drugs defined as essential medicines by the WHO.

Overall, 63% of reported drug shortages were branded drugs (424/671) and only 37% were generics. For 42% of these branded drugs (178/424), generics already entered the market while in the other 58% of the cases, no competitors were marketed yet. At the same time, 76% of permanent shortages (117/154) affected branded drugs. For essential drugs, 55% of reported drugs in shortage (110/200) were branded drugs. For oncology drugs 46% of the shortages affected branded drugs (33/71).

**Figure 4:**
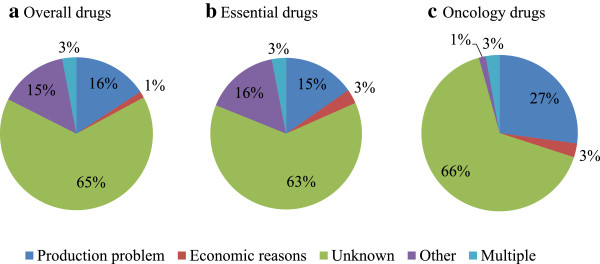
**Proportion of the reported drugs per cause for drug shortages.** The proportion of drugs per cause is shown for **a)** overall drugs (n = 171), **b)** essential drugs (n = 200) and **c)** oncology drugs (n = 71).

The cause of the drug shortage was only specified in 36% of the cases (236/671). In order to increase homogeneity in the data obtained from different reporting systems, reported causes were classified in five broad groups i.e. production problems, economic reasons, other causes, multiple reasons and unknown cause (Table 2). When the cause for the shortage was reported, production problems and causes defined under the group “other” were leading for overall shortages as well as for shortages of essential medicines (Figure 4a and 4b). For oncology drugs, shortages were mostly due to production problems and commercial reasons however the number of observations was very limited (Figure 4c). The class of production problems presents technical problems, quality related issues and shortage of active pharmaceutical ingredients (Table 2). However not all reporting systems distinguished between these different production problems (Table 2). The group defined under “other causes” included a variety of causes that were only reported a single time or were only reported in case of one country (Table 2). The majority of causes defined under “other” presented regulatory problems reported by Italy. Although EMA reported only a small proportion of the drug shortages considered in the national reporting systems, the causes behind the shortage were however more unfolded compared to the national reporting systems. Production problems were described as manufacturing defects that prevent the patients to inject the full dose, shutdown of production site, manufacturing problems leading to quality issues, shutdown of filling line and quality batched leading to a recall of several batches and stopping of production. Other causes mentioned by EMA are delay caused by accidental spillage and capacity problems at the laboratory were the drug is manufactured.

**Figure 5:**
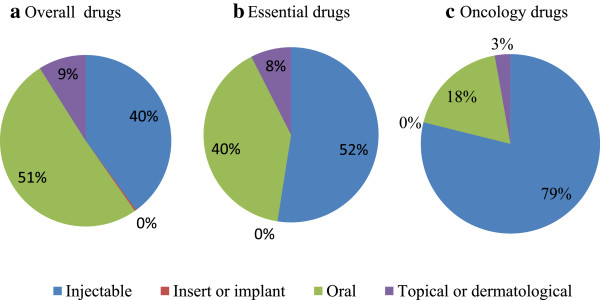
**Proportion of the reported drugs per route of administration.** The proportion of drugs per route of administration is shown for **a)** overall drug (n = 671), **b)** essential drugs (n = 200) and **c)** oncology drugs (n = 71).

The majority of reported drugs in short supply were oral drugs, followed by injectables (Figure 5a). For essential drugs and oncology drugs in short supply, the majority of reported drugs were injectables, followed by oral drugs (Figure 5b-c).

**Table 2:** **Causes for drug shortages reported in national reporting systems**

**Production problems**	
Italy	Production problem (at European level)
France	Problems with production
	Impurities found in syringe
	Lack of active substance
	Contamination particular lots
Germany	Production problem
	Technical defect in the production procces
	Problems with manufacturing the solvent
	Inability to meet the demand due to problem GMP compliance
Belgium	Production problem
	Re-engineering of the process and equipment at sterile production site
	Availability of raw material
The Netherlands	Production problems
	Delay startup new production line
	Problems with raw materials
	Recall of a batch
	Problems with quality
	Possible contamination
England	Manufacturing issues
Spain	Problems with sterility based on inspection in France and UK
**Economic reason**	
Italy	Commercial problem
Belgium	Stop due to commercial reasons
The Netherlands	Economic reasons
**Other**	
Italy	Administrative problem
	Natural disaster
	Regulatory problem
France	Mistake in labeling
England	Shortage of active pharmaceutical ingredient
	Stop supply of alternative
Spain	Marketing authorization cancelled
Belgium	Limited stock because unavailability of alternative product
	Problem of stock
The Netherlands	Increased demand
	Delayed supply
	Logistic problems
	Moving production sites
	Extensive controll
	Delay in delivery of new packages

## Discussion

This study investigated drug shortage reporting systems from seven European countries, in order to collect and present data about drugs in short supply in Europe. The results characterized drug shortages in European countries as oral, branded drugs that affect a broad range of disease domains. Causes for drug shortages were widely underreported in European countries; however production problems in a broad sense were suggested to be the major cause.

These results are based on eight different reporting systems presenting shortages in seven countries. Drug shortages are a dynamic problem and databases need regular updates, so data included in this study can present only a snapshot of the problem. Further, each system applies its own standards for reporting shortages so reporting bias needs to be considered with regard to the results of this study. First, reporting systems each have a different scope. The scope of the Italian reporting system is the broadest compared to other systems considered in this study, since there are no limitations with regard to the type of drugs included in the reporting system, and all stakeholders of the health care system are eligible for reporting. Only a limited proportion of the reported shortages were mentioned in advance, complicating early anticipation by health care workers and jeopardizing patient care. For the cause of drug shortages, it is unclear whether particular causes do not occur in a country or if the reporting system is not sufficiently specified to identify these causes. Causes of drug shortage are categorized in broad groups and could only be analyzed summarily in this study. Considering drug shortages for which information about the cause is available, production problems take the lead. It is generally assumed that injectables are most susceptible to production problems and quality defects compared to other formulations, due to the complexity of the manufacturing process [5, 17]. In U.S. this is believed to be related to economic issues, such as price competition for generic injectables, which reduces profitability and market attractiveness and discourages investments in quality of generics by the manufacturer [1, 6, 7, 17]. While the theory of economic root causes behind drug shortages was first only hypothesized in scientific literature [5, 6, 17], it is recently recognized in the revised action plan against drug shortages by the FDA in the U.S., published in October 2013 [18, 19]. In Europe however, no efforts are yet made to unveil the root causes for drug shortages [18, 20]. Economic root causes behind drug shortages are supposedly also not reported because it can lead to a negative perception about the marketing authorization holder. Moreover, drug shortages in considered European countries were not affecting generic drugs in the majority of reported causes although this observation may be biased since some of the considered reporting systems limit their data to drugs without alternative treatments. When however focusing on essential medicines and oncology drugs, the importance of generic injectables in shortages increased. The larger share of branded drugs in European drug shortages may be explained by international reference pricing applied in a number of European countries, proposed to have a downwards effect on prices for branded products [20]. However, particular regulatory frameworks present at national and European level are potential candidates for the root cause of production problems. Capacity problems can be caused by tendering procedures, which intend to grant the most favorable reimbursement for those products that are offered at the lowest price during a public procurement and are widely applied in European countries. In some cases supply is even reserved for products that won the tender, and the marketing of the drug becomes less profitable and less attractive for manufacturers who did not win. When the number of suppliers is limited or prices are set unattainably low, tendering will reduce the capacity of the market to step up supply if needed and pave the way to drug shortages [21]. Further, some manufacturers manage and restrict supply to wholesalers by introduction of supply quotas with the aim to ensure patient access and obtain their market share. Supply quotas are currently more under the suspicion of causing drug shortages instead of avoiding them because manufacturers may not be able to cope with an (unexpected) increase in demand [20]. In order to ensure sustainable drug supply, the allocation of quotas must be based on reliable data and able to cope with fluctuations in demand instead of being based on historic data, as mostly is the case [20]. Also interaction between different European member states can occur and affect the drug supply chain. The European Union is a single market with free movement of goods. Intellectual property rights are exhausted once a product has been put on the market with permission of the patent holder. This allows wholesalers to buy products in countries with low prices and sell them at high margins in countries with high price for the particular drug. This phenomenon of parallel trade can effectuate drug shortage when export of the product is not foreseen and cannot be anticipated [22]. Additionally, when imported products are sold cheaper than prices valid in that country, marketing of the product by national suppliers can also be discouraged [22]. The impact of parallel trade on drug shortages is poorly studied but heterogeneous price setting between European countries makes its role in drug shortages plausible.

Further understanding of the drug shortage problem in Europe however requires improved reporting systems that allow insights in the reason for shortages. National health authorities need to follow-up the consequence of national policies on the sustainability of the market and the effect of interactions between countries on national drug shortages needs to be monitored at European level. The European Medicines Agency (EMA) already reported about drug shortages in the past although publically available information was restricted, incomplete and limited to a small number of drugs, illustrating the need for a profound European initiative.

## Conclusions

Drug shortages are a global and increasing problem. Production problems seem the leading cause of shortages in European countries and U.S. but the characteristics of drugs in shortage can differ between these two regions. A strong link between production problems and market attractiveness is proposed and this needs to be investigated in Europe. Therefore, more profound insights in the underlying causes are required. Cost-containment measures need to be applied with scrutiny to ensure that all players of the drug supply chain can undertake their activities in a sustainable way. Therefore, national health authorities need to monitor the impact of national policies concerning price and reimbursement on availability of drugs. Additionally, further initiatives at European levels are required to monitor the interaction between national European markets. In that way, the lack of market capacity and reduced market attractiveness from the perspective of every player in the drug supply chain can be timely traced and anticipated and a fundamental solution for drug shortages can be developed, and this with the aim of guaranteeing the availability of medicinal products to the patient.

## Abbreviations

U.S.: United States
FDA: Food and Drug Administration
ASHP: American Society of Health-System Pharmacists
WHO: World Health Organization
EMA: European Medicines Agency
EGA: European Generics Association
EAHP: European Association of Hospital Pharmacists
GIRP: European Association of Pharmaceutical Full-line Wholesalers
ISPOR: International Society for Pharmaco Economics and Outcomes Research
EFPIA: European federation pharmaceutical industry associations
ATC: Anatomic Therapeutic Chemical Classification System
MMA: Medicare modernization act.
